# Nitidine chloride prevents OVX-induced bone loss *via* suppressing NFATc1-mediated osteoclast differentiation

**DOI:** 10.1038/srep36662

**Published:** 2016-11-08

**Authors:** Qian Liu, Tao Wang, Lin Zhou, Fangming Song, An Qin, Hao Tian Feng, Xi Xi Lin, Zhen Lin, Jin Bo Yuan, Jennifer Tickner, Hua Gang Liu, Ming Hao Zheng, Jiake Xu, Jin Min Zhao

**Affiliations:** 1 Research Centre for Regenerative Medicine, Department of Trauma Orthopedic and Hand Surgery, The First Affiliated Hospital of Guangxi Medical University, Guangxi, 530021, China; 2 School of Pathology and Laboratory Medicine, The University of Western Australia, Perth WA 6009, Australia; 3 Department of Orthopedics, The First Affiliated Hospital of Gaungxi Medical University, 22 Shuangyong Road, Guangxi, 530022, China; 4 School of Pharmacology, Guangxi Medical University, Guangxi, 530021, China; 5 Shanghai Key Laboratory of Orthopaedic Implant, Department of Orthopaedics, Shanghai Jiao Tong University School of Medicine, Ninth People’s Hospital, Shanghai, 200011, China; 6 Program of Nutrition and Bone & Joint Health, Nestlé R&D (China) Ltd. Building 5, No. 5 Dijin Road, Haidian District, Beijing, 100095, China; 7 Centre for Orthopaedic Research, School of Surgery, The University of Western Australia, Perth WA 6009, Australia; 8 Guangxi Key Laboratory of Regenerative Medicine, Guangxi Medical University, Guangxi, 530021, China

## Abstract

Nitidine chloride (NC), a bioactive alkaloid isolated from *Zanthoxylum nitidum*, has been used as a herbal ingredient in toothpaste that prevents cavities for decades. It also displays potential antitumor and anti-inflammation properties. However, its anticatabolic effect on bone is not known. We investigated the effect of NC on osteoclastogenesis, bone resorption and RANKL-induced NF-κB and NFATc1 signalling. In mouse-derived bone marrow monocytes (BMMs), NC suppressed RANKL-induced multinucleated tartrate-resistant acid phosphatase (TRAP)-positive osteoclast formation and bone resorption in a dose dependent manner. NC attenuated the expression of osteoclast marker genes including cathepsin K, D2, calcitonin receptor, NFATc1, and TRAP. Further, NC inhibited RANKL-activated NF-κB and NFATc1 signalling pathways. *In vivo* study revealed that NC abrogated oestrogen deficiency-induced bone loss in ovariectomized mice. Histological analysis showed that the number of osteoclasts was significantly lower in NC-treated groups. Collectively, our data demonstrate that NC suppressed osteoclastogenesis and prevented OVX-induced bone loss by inhibiting RANKL-induced NF-κB and NFATc1 signalling pathways. NC may be a natural and novel treatment for osteoclast-related bone lytic diseases.

Bone is a dynamic tissue constantly undergoing remodelling by two opposite forces, namely bone formation by osteoblasts and bone resorption by osteoclasts. Keeping the balance between bone formation and bone resorption is essential to maintain bone homeostasis. Bone resorption surpassing bone formation results in osteoporosis, characterized by loss of bone mass and architectural deterioration of the skeleton.

Bone resorbing osteoclasts are unique multinucleated TRAP-positive cells, derived from the bone marrow macrophage and monocyte lineages. Receptor activator of NF-κB ligand (RANKL) is a member of the tumour necrosis factor family (TNF) expressed by osteoblasts, chondrocytes, and osteocytes and is essential for osteoclastogenesis^1^. RANKL interacts with its receptor RANK to activate a cascade of intracellular signalling pathways, including nuclear factor-κB (NF-κB), mitogen activated protein kinase (MAPK), nuclear factor of activated T-cells (NFAT), Akt, and calcium/calmodulin-dependent kinase pathways. NF-κB signalling pathways play a key role in osteoclast formation^2^,^3^. RANKL-induced activation of the nuclear factor of activated T-cells cytoplasmic 1 (NFATc1) signalling pathway also represents a master switch for regulating terminal differentiation of osteoclasts^4^. Further, an antagonist targeting TRAF6, a RANK signalling adaptor molecule, has been shown to be effective at inhibiting osteoclastogenesis^5^. Therefore, targeting RANKL-activated downstream signalling pathways is a promising strategy to inhibit excessive bone resorption, thereby alleviating bone loss.

NC, a benzophenanthridine alkaloid isolated from *Zanthoxylum nitidum* (Rutacease) and *Fagara zanthoxyloide*^6^, has been used as a herbal ingredient in toothpaste that prevents cavities for decades. Recent studies have shown that NC has antitumor and anti-inflammation properties. NC inhibited breast cancer cell migration and invasion by suppressing gene expression of matrix metalloproteases (MMP)-9 and MMP-2 and blocking the c-Src/FAK signalling pathway^7^. NC also inhibited renal cancer cell metastasis by suppressing AKT and ERK signalling pathways^8^,^9^. Interestingly, NC exerted its anti-inflammatory effects by modulating MAPK and NF-κB pathways and subsequently suppressing lipopolysaccharide (LPS)-induced TNF-α, IL-1β, and IL-6 production in RAW 264.7 cells^10^. However, until now there has not been any report on the effect of NC on osteoclastogenesis and bone resorption.

In this study, we first examined the effects of NC on osteoclastogenesis and bone resorption *in vitro*. We found that NC dose dependently suppressed RANKL-induced osteoclast differentiation and osteoclastic bone resorption. Additionally, we found that NC inhibited RANKL activated NF-κB and NFAT signalling pathways. To further examine the physiological relevance of NC’s inhibitory effect on osteoclastogenesis and bone resorption, we used an OVX mouse model to examine whether NC had a preventative effect on oestrogen deficiency-induced bone loss *in vivo*. In line with our *in vitro* results, NC treatment significantly prevented OVX induced bone loss and abrogated excessive osteoclast formation. Our data clearly show that NC suppresses osteoclastogenesis and osteoclastic bone resorbing activity through inhibition of RANKL-induced NF-κB and NFAT signalling pathways.

## Results

### The effect of NC on RANKL-induced osteoclastogenesis, and osteoclast viability and apoptosis

**Figure 1:**
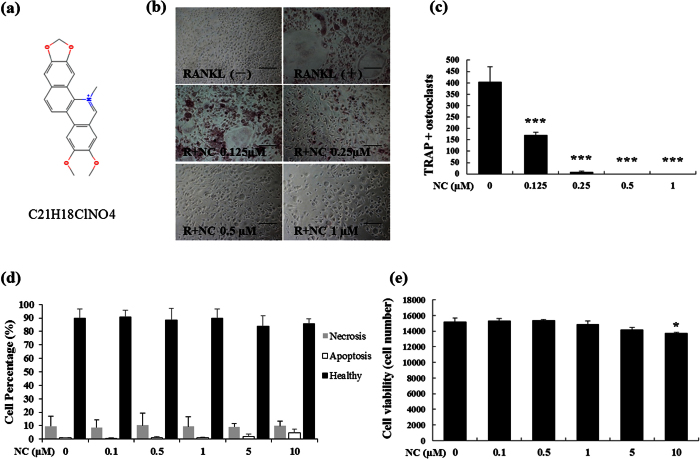
The effect of NC on RANKL-induced osteoclast formation and osteoclast apoptosis and viability. (**a**) Chemical structure of NC (PubChem substance ID 12013221). (**b**) BMM cells were cultured in the presence of M-CSF and RANKL (100 ng/ml) with or without varying doses of NC for 5 days and stained for TRAP expression. Light microscope images depicting the dose-dependent effect of NC on RANKL-induced osteoclastogenesis. (**c**) TRAP-positive multinuclear cells containing three or more nuclei were scored. (**d**) The effects of different doses of NC on apoptosis were detected through Annexin V staining and flow cytometry. (**e**) Cells were treated with different does of NC for 48 hours and cell viability was measured by MTS assay. (*p value < 0.05, ***p value < 0.001).

To examine the effect of NC (Fig. 1a) on osteoclastogenesis, BMMs (isolated from the long bone of wild type mice) were treated with 100 ng/mL RANKL and 10 ng/mL MCSF (macrophage colony stimulating factor) in the presence of various concentrations of NC. The results showed that osteoclast formation decreased with increasing concentrations of NC (Fig. 1b). The total number of multinucleated TRAP-positive cells was significantly lower as NC concentration increased from 0.125 μM to 1 μM NC relative to the control group (Fig. 1c). No osteoclasts were observed at doses of NC higher than 0.5 μM (Fig. 1b). These results suggest that NC dose-dependently inhibits RANKL-induced osteoclastogenesis in BMM cells.

To examine the induction of apoptosis with NC, cells were incubated with various doses of NC and then stained with Annexin V and PI. Increasing concentrations of NC did not induce either necrosis or apoptosis pathways in RAW 264.7 cells (Fig. 1d). To further examine the effect of NC on osteoclast viability, MTS assay was used to determine the effect of NC doses on BMM numbers. Figure 1e shows that cell viability did not change in the presence of NC, up to 5 μM concentration. These results indicate that NC profoundly inhibits osteoclast formation but does not affect osteoclast viability or apoptosis at concentrations under 5 μM.

To examine the effect of NC on cytoskeleton of osteoclasts, we tested whether F-actin structures were affected by NC. Osteoclasts cultures treated with or without NC were double stained with rhodamine-conjugated phalloidin and DAPI, and then visualized by confocal microscope. The results showed that NC untreated cells had typical osteoclasts morphology, including the formation of F-actin ring and numerous nuclei. In contrast, NC treated cells had smaller size and fewer nuclei numbers with poor or disrupted F-actin ring formation (Supplementary Fig. S1).

### The effect of NC on RANKL-induced expression of osteoclast marker genes

**Figure 2:**
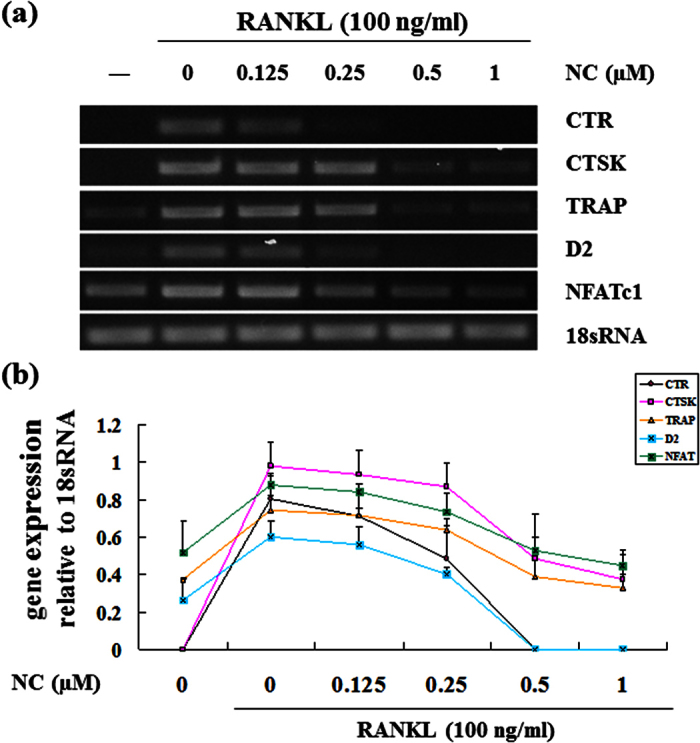
Suppression of RANKL-induced osteoclast gene expression by NC. BMMs were seeded in the presence and absence of 100 ng/ml of RANKL with different dose of NC (0, 0.125, 0.25, 0.5 and 1 μM) for 7 days. (**a**) The mRNA expression levels of the indicated genes were determined by RT-PCR. (**b**) Plot of gene expression level relative to NC concentration, normalised to 18sRNA.

Next, we examined the effect of NC on the expression of osteoclast marker genes. For BMM-derived osteoclasts, BMM cultures were treated with M-CSF and RANKL in the absence or presence of NC for seven days. Gene expression of calcitonin receptor, cathepsin K, TRAP, and NFATc1 decreased in a dose dependent manner during RANKL-induced osteoclastogenesis (Fig. 2a,b). This is consistent with NC’s inhibitory effects on osteoclast differentiation. NC also suppressed the gene expression of the osteoclast fusion marker D2.

### The effect of NC on osteoclastic bone resorption

**Figure 3:**
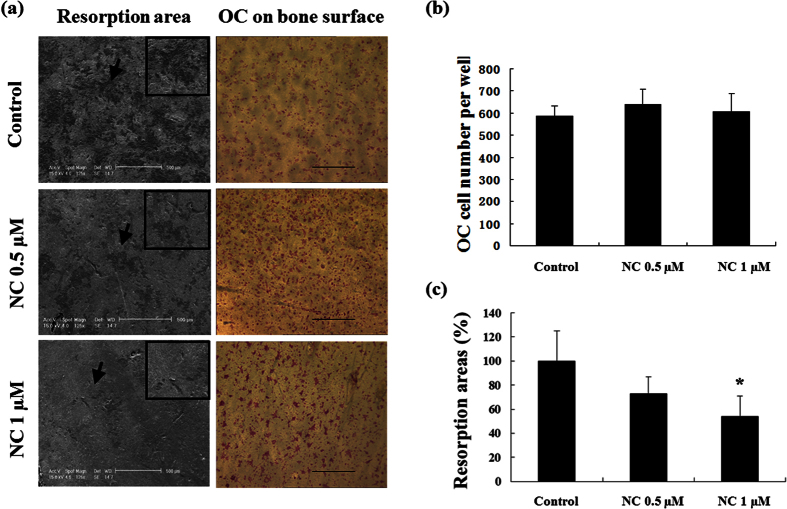
NC reduces bone resorption. Equal numbers of osteoclast-like cells derived from BMM cells were seeded onto bone slices and permitted to attach before exposure to NC at different doses (0, 0.5 and 1 μM) for 48 hours. (**a**) Representative SEM images of bone resorption pits (magnification 125x), arrows show inset area (magnification 500x), and TRAP staining on bone surfaces (magnification 40x). (**b**) Quantification of the effect of NC on the TRAP positive osteoclasts number. (**c**) The percentage area covered by resorption pits relative to untreated control was measured using ImageJ software. (*p value < 0.05).

To investigate the effect of NC on murine osteoclastic bone resorption, osteoclast-like cells were seeded on to bovine bone slices with culture medium and RANKL for 12 hours, followed by treatment without or with NC (0.5 or 1 μM) for a further 48 hours. The total number of TRAP-positive cells on each bone slice was not affected in the presence of NC (data not shown), while the osteoclastic bone resorption area decreased significantly at 1 μM NC (Fig. 3a). The morphology of the resorption pits in the NC treatment group was smaller and shallower compared to untreated controls. Figure 3b shows total number of osteoclasts, and Fig. 3c indicates the percentage areas of resorption pits. These results demonstrate that NC inhibits bone resorption without affecting osteoclast survival.

### The effect of NC on RANKL-induced NF-κB activation and protein expression

**Figure 4:**
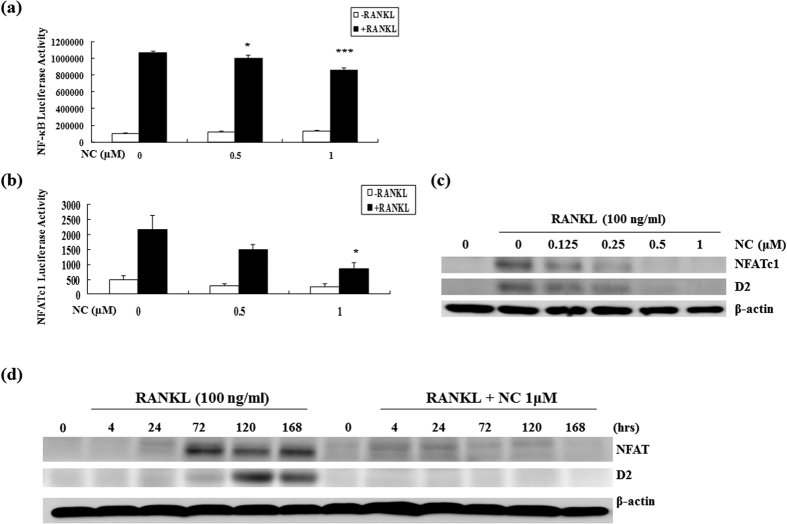
The effect of NC on RANKL-induced NF-κB and NFATc1 activities. (**a**) NF-κB activity was assessed by luciferase assay following pre-incubation of RAW 264.7 cells stably transfected with a 3κB-Luc-SV40 luciferase reporter gene with varying doses of NC followed by stimulation with RANKL for 8 hours. (**b**) RAW264.7 cells were transiently transfected with pNFATc1-TA-Luc vector and then treated with medium alone, RANKL (100 ng/ml), NC at 0.5 μM and 1 μM in the presence or absence of RANKL for 24 hours and NFATc1 luciferase activity was measured. (*p < 0.05 vs. control) (**c**) BMMs were incubated with different doses of NC for 1 hour and treated with 100 ng/ml RANKL for 7 days and NFATc1 and D2 protein levels assessed. (**d**) BMMs were incubated with 1 μM NC for 1 hour and treated with RANKL for the indicated times. The β-actin blot is shown as a loading control. (*p value < 0.05, ***p value < 0.001). Note that NFATc1, D2 and β-actin protein blot slabs were cropped from full blots with the corresponding well established sizes of NFATc1, D2 and β-actin proteins.

To examine the role of NC on RANKL signalling, we next tested the effect of NC on RANKL-induced activation of NF-κB. RAW264.7 cells, stably transfected with the 3kB-Luc-SV40 reporter gene were pre-incubated with varying doses of NC for one hour. Luciferase activity in the lysate was examined after eight hours of stimulation with 100 ng/mL of RANKL. Our results demonstrated that NC suppressed RANKL-induced NF-κB activation at 0.5 μM and 1 μM in a dose dependent fashion (Fig. 4a). The inhibitory action of NC on RANKL-induced NF-κB activity was significantly higher as the NC concentration increased from 0.5 μM to 1 μM.

To further evaluate the upstream pathway resulting in NF-κB activation, we tested the effect of NC on IκBα degradation by RANKL. BMMs were pre-treated with 1 μM NC for one hour, and then stimulated with 100 ng/mL RANKL for varying time periods. Western blotting showed that NC had little effect on RANKL-induced degradation of IκBα (Supplementary Fig. S2). We also examined the effect of NC on nuclear translocation of p65 in BMMs. The pretreatment of NC (1 μM) for 1 hour also had little effect on RANKL-induced p65 nuclear translocation in BMMs (Supplementary Fig. S3).

### NC suppresses RANKL-induced NFATc1 activation and protein expression

To further examine the role of NC on RANKL signalling, we first tested its effect on RANKL-induced activation of NFATc1. RAW 264.7 cells were transiently transfected with a pNFATc1-TA-Luc vector. Cells were treated with NC at 0.5 μM and 1 μM in the presence or absence of RANKL (100 ng/mL). Luciferase activity was measured after 24 hours of treatment. As shown in Fig. 4b, NC suppressed RANKL-induced NFATc1 activation in a dose dependent manner. Next, we examined whether NC suppresses RANKL-induced NFATc1 protein expression using a range of NC doses. BMMs (1 × 10^6^) were incubated with different doses of NC for one hour and then treated with 100 ng/mL RANKL for seven days in 6-well plates. Consistent with the observed reduction in NFATc1 activation western blot analysis indicated that NC inhibited osteoclast formation by suppressing the RANKL-induced NFATc1 signalling pathway. Both NFATc1 and D2 (Fig. 4c) protein levels were inhibited in a dose dependent manner. Analysis of temporal regulation of NFATc1 and D2 expression was performed using long time course western blots. In the absence of NC, both NFATc1 and D2 protein expression were highest at 120 hours (day 5) and began to decline afterwards. The NFATc1 and D2 expression levels were low to undetectable at all-time points in the presence of 1 μM NC (Fig. 4d).

### NC protects against OVX-induced bone loss in mice

**Figure 5:**
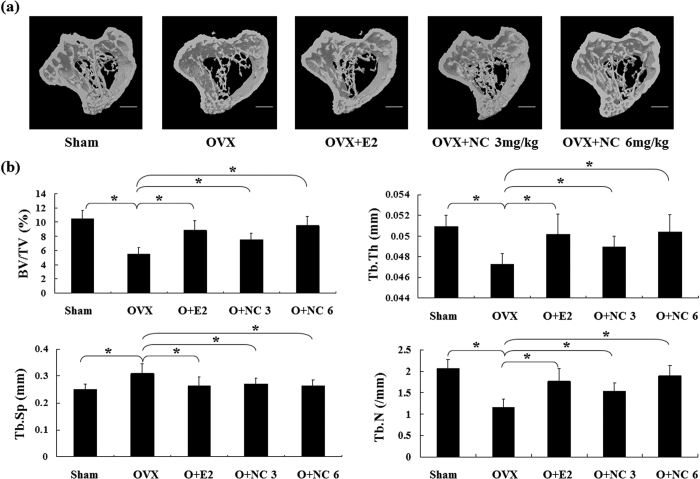
NC prevented bone loss in OVX C57BL/6J mice *in vivo*. (**a**) Trabecular bone microarchitecture by 3D micro-CT analysis in the proximal tibial metaphysis. Trabecular bone was analysed by micro-CT in sham-operated or ovariectomized mice after vehicle, E2 (50 ng/kg) or NC treatment (3 and 6 mg/kg). (**b**) Bone volume/tissue volume (BV/TV), trabecular space (Tb. Sp), trabecular thickness (Tb. Th) and trabecular number (Tb. N). Values are means ± SE from 6 mice per group. (*p < 0.05 vs. control).

The OVX mouse model of postmenopausal bone loss was used to examine the effects of NC on osteolysis *in vivo*. C57BL/6J mice were either ovariectomized or sham-operated. After 1 week recovery time OVX mice were treated with either vehicle (n = 6), oestrogen (E2) (50 ng/kg; n = 6), or NC (3 or 6 mg/kg/2 days; n = 6), for six weeks and sacrificed at the end of treatment. Mouse tibias were removed and subjected to micro-CT assessment (Fig. 5a). Micro-CT analysis showed that OVX induced a significant bone loss, and that treatment with oestrogen rescued the bone loss associated with OVX. Treatment with NC at 3 and 6 mg/kg doses was also able to prevent the OVX-induced reduction in trabecular bone volume, trabecular thickness, and trabecular number and the OVX-induced increase in trabecular separation (Fig. 5b). Furthermore, the 6 mg/kg dose was as effective as oestrogen in protecting against bone loss in OVX treated mice.

**Figure 6:**
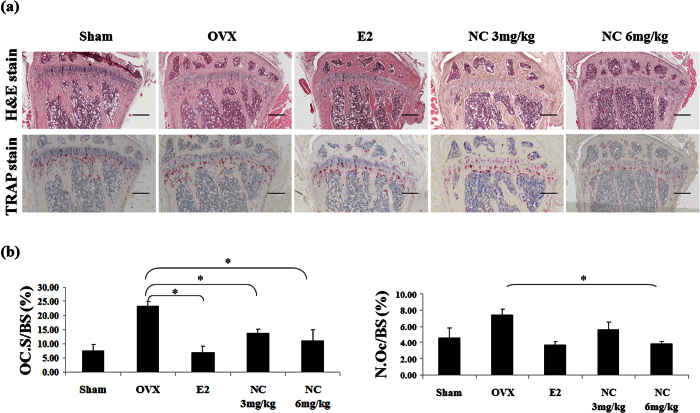
Histological analysis of trabecular bone with H&E and TRAP staining in proximal tibia. (**a**) representative images of H&E and TRAP staining (×40 magnification). (**b**) Oc.S/BS, osteoclast surface per bone surface; N.Oc/BS, number of osteoclasts per bone perimeter. Values are means ± SE from 6 mice per group. *p < 0.05 compared with the sham group.

Consistent with the observed effects of NC on bone resorption *in vitro,* histomorphometric analysis of the bones of OVX mice showed reduced osteoclast surface/bone surface (OcS/BS) and number of osteoclasts in NC treated groups (Fig. 6a,b). Treatment with 6 mg/kg NC decreased OVX-induced bone loss through a reduction in osteoclast numbers to a similar extent as was observed in estrogen treated controls.

## Discussion

Abnormal osteoclast formation and excessive osteoclastic bone resorption result in pathologic bone loss in diseases. RANKL activates downstream pathways including NF-κB and NFATc1 that are critical for osteoclast formation and activity^11^. We have screened and identified several natural compounds that exhibit inhibitory effects on osteoclast formation and bone resorption, such as mangiferin^12^, naringin^13^, and NC. These natural compounds have similar biological effects on anti-cancer or anti-inflammatory activities through the regulation of NF-κB^14^,^15^,^16^,^17^. Recently, the chemical composition of NC has been analysed, but its role in osteoclastogenesis is still unclear.

In this study, we report that NC inhibits osteoclast differentiation and bone resorption. Consistent with its inhibitory effect on osteoclastogenesis, NC also suppresses osteoclast marker gene expression, including calcitonin receptor, cathepsin K, TRAP, NFATc1, and D2. BMM cells were used to define the direct role of NC on RANKL-induced osteoclastogenesis. The engagement of RANKL-RANK-TRAF6 will lead to activation of downstream signalling pathways, including NF-κB, NFAT, and MAPK^18^. We have also investigated the role of NC on RANKL signalling pathways, including NFAT, NF-κB, and MAP kinases (MAPK results not shown). Of particular importance, we have found that NC inhibits RANKL-induced NFAT protein expression and activation, whilst having limited effects on NF-κB and MAP kinase pathways. Collectively, our results indicate that the inhibitory effects of NC are predominantly mediated through the regulation of NFAT and NF-κB activity.

To date, our knowledge regarding the role of NC in cell signalling is very limited. Only recently was NC found to inhibit the phosphorylation of c-Src, FAK, MAPKs, and inhibit the activation of RhoA, Rac1, and AP-1 transcriptional activity induced by PDGF in breast cancer cells^7^. However, we found that NC has little effect on calcineurin protein expression and RANKL-induced c-Src phosphorylation (data not shown). Other studies have shown that NC induces the activation of the caspase-dependent pathway in MG63, an osteosarcoma cell line^13^. Overall, these data provide a clue to the mechanistic explanation for the inhibitory effects of NC on osteoclast formation and bone resorption. It remains to be determined whether NC could influence these signalling pathways that also contribute to osteoclast formation and bone resorption.

The *in vitro* inhibition of osteoclastogenesis and bone resorption by NC also correlates with its *in vivo* effect of protecting against osteolysis in an OVX mouse model. These results suggest that NC may have therapeutic potential for the treatment of osteoclast-related bone diseases by targeting osteoclasts. However, the role of NC in osteoblast differentiation and mineralization is unknown. The maintenance of dynamic bone homeostasis involves both bone resorption by osteoclasts and bone synthesis by osteoblasts^19^. Thus, it will be interesting to test the effects of NC on the osteoblastic lineage in our future experiments.

Taken together, this study demonstrates that NC displays an inhibitory effect on RANKL-induced osteoclast differentiation and bone resorption *in vitro* and osteolysis *in vivo*. Defining the precise target of NC in signalling for osteoclast differentiation and function will require further investigation of the downstream RANKL-induced signalling pathways in osteoclasts. Dissecting the mechanism of action of NC at the molecular and cellular level will provide very important information for its potential use in the treatment of osteoporosis.

## Methods

### Reagents

NC was purchased from the National Institute for Control of Pharmaceutical and Biological Products (Beijing, China). RAW 264.7 cells were ordered from the American Type Culture Collection (Rockville, MD, USA). Murine M-CSF (mM-CSF) was obtained from R&D Systems, (Minneapolis, USA). Alpha Modified Minimal Essential Medium (α-MEM) was purchased from Thermo Fisher (Sydney, Australia) and Fetal Bovine Serum (FBS) from TRACE (Sydney, Australia). Recombinant GST-rRANKL protein was expressed and purified as previously described^20^. Antibodies to IκBα and NFATc1 were obtained from Santa Cruz Biotechnology Com. (CA, USA). Antibody to V-ATPase d2 was generated as previously reported^21^.

### Ethical Use of Animals

All procedures utilizing mice were performed in accordance with the National Health and Medical Research Council ‘Guidelines to promote the wellbeing of animals used for scientific purposes: The assessment and alleviation of pain and distress in research animals (2008)’, in conjunction with the ‘Australian code for the care and use of animals for scientific purposes 8th edition (2013)’; and also in accordance with the guidelines for ‘Ethical Conduct in the Care and Use of Nonhuman Animals in Research’ by the American Psychological Association. All protocols were approved by the UWA animal ethics committee (approval RA/3/100/1244) or the Animal Care and Welfare Committee of Guangxi Medical University (SYXK2009-0004) as indicated.

### *In vitro* osteoclast cell culture of bone marrow macrophage (BMM)

For *in vitro* osteoclast culture bone marrow macrophages (BMMs) were isolated from the bones of C57BL/6J mice, following protocols approved by the UWA animal ethics committee (approval RA/3/100/1244). These cells were cultured in α-MEM supplemented with 10% FBS, 5 μg/mL Penicillin, 50 U/mL streptomycin and 2 mM L-glutamine (complete MEM). Following isolation cells were cultured in complete MEM supplemented with 10 ng/mL of mM-CSF. Confluent cells were seeded into 96-well plates at a density of 8 × 10^3^ BMM cells per well with NC in the presence of RANKL (100 ng/mL) for seven days with media replaced every two days. At the end of the culture period cells were fixed with 4% paraformaldehyde (PFA) for 20 minutes at room temperature and TRAP staining was performed. TRAP-positive multinucleated cells with more than three nuclei were counted as osteoclasts.

### MTS assay

The MTS assay was performed according to the manufacturer instructions (Promega, USA). Briefly, BMM cells were seeded at a density of 6 × 10^3^ per well on a 96-well plate for overnight incubation. The cells were then incubated with differing concentrations of NC (0.1, 0.5, 1, 5 and 10 μM) for 48 hours. MTS solution was then added to the cells and incubated for two hours. Absorbance was measured at 490 nm using an ELISA plate reader.

### Apoptosis assay

To examine the effects of NC on apoptosis, a commercially available Annexin V Apoptosis Detection Kit (eBioscience, CA, USA) was used according to the manufacturer’s instructions. 1 × 10^5^ RAW 264.7 cells were seeded in αMEM medium with 10% FBS in a 6-well plate overnight. Varying doses of NC were added to each well and cells incubated for a further 24 hours. Cells were harvested by incubation with Tryple (Invitrogen, CA, USA), washed once with PBS and 1 × binding buffer, and then resuspended at a density of 1 × 10^7^ per mL in 1 × binding buffer. The cell suspension (100 μL) was mixed with 5 μL fluorochrome-conjugated Annexin V and incubated for 10 minutes. Cells were then washed and resuspended again with 200 μL 1 × binding buffer and 5 μL propidium iodide. Cell suspensions were then put on ice and stored in the dark until analysis by flow cytometry.

### Immunofluorescence staining of F-actin and confocal microscopy

For immunofluorescence study, BMMs were cultured with RANKL (50 ng/ml) and 10 ng/mL of mM-CSF in the presence or absence of NC. Medium were replaced every two days, and after 5 days, cells were fixed with 4% PFA. Cells were then washed with 1 × PBS for three times and permeablised with 0.1% Trition X-100 for 5 min. After that, cells were blocked with 3% BSA for 30 min and washed with 1 × PBS containing 0.2% BSA (BSA-PBS). Cells were then stained with Rhodamine-Phalloidin (Invitrogen) for at least 1 hour, and then washed with 0.2% BSA-PBS and PBS each for 4 times. Then, cells were stained with DAPI (Santa Cruz Biotechnology, California, USA) for 5 min, and washed with 1 × PBS for 3 times. Cells were then mounted with Prolong Gold antifade (Invitrogen) mounting medium for confocal microscopy.

### Reverse transcription (RT)-PCR

BMM cells were seeded in 6-well plates at a density of 1 × 10^5^ per well. Cells were cultured with RANKL in the presence or absence of 0.5 μM or 1.0 μM NC for seven days. Total RNA was extracted using Trizol in accordance with the manufacturer’s protocol (Life technologies, USA). For reverse transcription, single-stranded cDNA was prepared from 2 μg of total RNA using reverse transcriptase with an oligo-dT primer. Each cDNA (1 μL) was subjected to PCR amplification using specific forward and reverse primers. 18sRNA was used as an internal control. Specific primers of the following genes were used for PCR amplification: 18sRNA (Forward: ACC ATA AAC GAT GCC GAC T; Reverse: TGT CAA TCC TGT CCG TGT C), Calcitonin Receptor (Forward: TGG TTG AGG TTG TGC CCA; Reverse: CTC GTG GGT TTG CCT CAT C), Cathepsin K (Forward: GGG AGA AAA ACC TGA AGC; Reverse: ATT CTG GGG ACT CAG AGC), V-ATPase d2 (D2) (Forward: GGA TCC GAA TTC ATG CTT GAG ACT GCA GAG; Reverse: GGT CTA GAT TAT AAA ATT GGA ATG TAG CT3), NFATc1 (mouse NFATc1) (Forward: CAA CGC CCT GAC CAC CGA TAG; Reverse: GGC TGC CTT CCG TCT CAT AGT), TRAP (Forward: TGT GGC CAT CTT TAT GCT; Reverse: GTC ATT TCT TTG GGG CTT).

### NFATc1 luciferase reporter gene assay

The transcriptional activity of NFATc1 was monitored using the NFATc1 luciferase reporter gene. Using the diethylaminoethyl-dextran method, RAW 264.7 cells were temporarily transfected with 0.457 μg/μL of pNFATc1-TA-Luc vector (Promega, Sydney, Australia) in Dulbecco’s modified eagle medium (DMEM)/4-(2-hydroxyethyl)-1-piperazineethanesulfonic acid medium. After transfection, cells were placed in a 24-well plate and cultured with complete DMEM for 18~24 hours and pre-treated with NC for one hour, followed by stimulation with 100 ng/mL mouse RANKL for about 24~36 hours. NFAT activation was determined after harvesting the cells through measurement of firefly luciferase activity using the Promega Luciferase Assay System.

### NF-κB luciferase reporter gene assay

To examine the transcriptional activity of NF-κB, RAW 264.7 cells stably transfected with a 3κB-Luc-SV40 luciferase reporter gene containing three κB sites from the interferon gene was used as previously described^22^. Cells were plated at 1 × 10^5^ cells/well in 24-well plates, and pre-treated with NC for one hour. After stimulating with 100 ng/mL mouse RANKL for eight hours, cells were immediately washed twice in cold 1 × PBS. Lysis buffer (100 μl) was added to each well for the luciferase assay. RAW 264.7 cells were harvested and the cell luciferase activity was measured using the Promega Luciferase Assay System, according to the manufacturer’s instructions (Promega, Sydney, Australia).

### Bone resorption pit assay

For BMM derived osteoclasts, BMM cells were isolated from the long bone of C57BL/6J mice and cultured with α-MEM supplemented with 100 ng/mL RANKL and 10 ng/mL mMCSF on collagen coated 6-well plates for three days. Once mature osteoclasts had formed they were detached from the plastic using cell dissociation solution, pelleted and resuspended, and 1 × 10^3^ were seeded onto ~0.75 mm thick bovine bone slices with complete α-MEM culture medium, mMCSF (10 ng/mL), and mRANKL (100 ng/mL) in a 96-well plate. Twelve hours after seeding, NC was added to the culture. After an additional 48 hours incubation at 37 °C, the cells were fixed with 4% PFA, stained with TRAP and counted under a light microscope. To remove the extraneous cells, the bone slices were gently brushed and sonicated. The resorption pits were examined using a Philips XL30 scanning electron microscope and the percentage of bone surface area resorbed per osteoclast was quantified using Scion Image software (Scion Corperation, National Institutes of Health)^23^.

### Western blot assay

Freshly isolated BMM cells were cultured in a 6-well plate. BMMs were pre-treated with different doses of NC (0.5 μM or 1 μM) for one hour, and then stimulated with RANKL for 0, 10, 20, 30, 60, 120 min in a short-time course western blot assay. For long time course assays BMMs were stimulated with RANKL for 0, 4, 24, 72, 120, or 168 hours. Cells were then lysed for protein extraction using ice cold RIPA buffer. SDS-PAGE was performed and proteins were transferred to a nitrocellulose membrane. The membrane was blocked using 5% w/v skim milk in TBS-Tween solution for at least one hour. After washing with TBS-Tween, antibodies (IκBα, D2, and NFATc1) were used to detect RANKL-induced signalling pathways. The immunoreactivity was visualized using Enhanced Chemiluminescence (ECL) reagents (Perkin Elmer, Waltham, MA, USA).

### p65 immunohistochemistry

BMMs were seeded into 96-well plates at the density of 2 × 10^4^/well and then incubated at 37 °C overnight. The next day, cells were first pre-incubated with NC (1 μM) for 1 h, then stimulated with RANKL (100 ng/mL) for 30 min. The cells were then washed once with 1 × PBS, then fixed with 4% PFA for 10 min. After washed three more times with 1 × PBS, cells were permeablized for 5 min with 0.1% Triton X-100 in PBS. After that, cells were washed twice with 0.1% BSA-PBS, cells were incubated at 37 °C for 45 min with anti-p65 antibody (50 μl/well; Santa Cruz Biotechnology, Inc., CA, USA) diluted 1:200 in 0.1% BSA-PBS. The cells were then washed four times with 0.1% BSA-PBS, four times with 1 × PBS, once with 0.1% BSA-PBS before the addition of streptavidin-horseradish peroxidase (Dako, Victoria, Australia). After incubation for 20 min at room temperature and washing for four times with 0.1% BSA-PBS, four times with PBS, once with 0.1% BSA-PBS Dako Liquid DAB (20 μL/well; Dako, Victoria, Australia) was added to the plates for up to 30 min or until brown color presented.

### Ovariectomy (OVX) animal model

Thirty C57BL/6J female mice were housed in a specific pathogen free animal laboratory for a week to acclimatise. C57BL/6J mice handling procedures were carried out in accordance with the guidelines for Ethical Conduct in the Care and Use of Nonhuman Animals in Research by the American Psychological Association and were approved by The Guangxi Medical University Ethics Committee. The experimental procedures were approved by the Animal Care and Welfare Committee of Guangxi Medical University (SYXK2009-0004). Thirty female C57BL/6J mice aged 8 weeks were randomly assigned to five groups of 6 each. The mice were anesthetized with 10% chloral hydrate solution and each side of ovary was removed. One week after surgery, mice were given intraperitoneal (i.p.) injection of NC (3 mg/kg; 6 mg/kg) in 1% DMSO in physiological saline every two days for six weeks. The negative control group (OVX group) mice received 10% DMSO (in physiological saline) as a placebo. The positive control group (E2 group) mice received 50ng/kg E2. At six weeks post-treatment, the mice were euthanized. The tibias were removed, fixed in 4% PFA for 24 hours and radiologically analysed using micro-computed tomography (micro-CT).

### Micro-CT analysis

The tibias were washed three times with 1 × PBS and then immobilized in a tube for scanning. Bones were scanned at 9 μm resolution in a Skyscan 1176 micro-CT instrument (Skyscan, Aartselaar, Belgium). The source voltage used was 50 kV, current 500 μA, and the filter was 0.5 mm aluminium. Scans were reconstructed using NRecon software (cone beam reconstruction algorithm, Skyscan) with a constant global threshold. Reconstructed samples were then analysed using CTAn software (Skyscan), with trabecular and cortical regions of interest determined in reference to the bottom of the growth plate. Trabecular bone was analysed within a volume of slices spanning a 1-mm distance, starting 0.5 mm from the bottom of the growth plate. The trabecular regions of interest were outlined by interpolation of operator-drawn regions exclusively representing trabecular bones. Regions of interest were binarised using constant threshold values and analysed using CTAn software.

### Bone histomorphometric analysis

Tibias were fixed in 4% PFA, then washed three times with 1 × PBS. After that, tibias were decalcified with 14% EDTA for 7 days. Samples were then embedded in paraffin for HE or for the enzymatic activity of tartrate resistant acid phosphatase (TRAP). Bone histomorphometric analysis was performed by quantifying parameters including osteoclast surface per bone surface (OcS/BS) and number of osteoclasts per bone perimeter (N.Oc/BS) using BIOQUANT OSTEO software (BIOQUANT OSTEO 2013 Ver. 13.20.6, Nashville, USA). For all analysis an area 1 mm in height, 0.5 mm below the growth plate and excluding cortical bone was analysed.

### Statistical analysis

All data presented in this study are representative of one of three independent experiments, and the results are presented as mean ± SEM. For quantitative analyses of bone volume/total volume (BV/TV), trabecular separation (Tb.Sp), trabecular number (Tb.N), and trabecular thickness (Tb.Th), osteoclast surface/bone surface (Oc.S/BS), osteoclast number/bone surface (N.Oc/BS), Values are means ± SE from 6 mice per group. Statistical analyses included paired or unpaired Student’s t-tests using Microsoft Excel 2003. A p-value of <0.05 was set as statistically significant.

## Additional Information

**How to cite this article**: Liu, Q. *et al*. Nitidine chloride prevents OVX-induced bone loss *via* suppressing NFATc1-mediated osteoclast differentiation. *Sci. Rep.* **6**, 36662; doi: 10.1038/srep36662 (2016).

## Supplementary Material

Supplementary Information
