# Supplementary material for: Nitidine chloride prevents OVX-induced bone loss via suppressing NFATc1-mediated osteoclast differentiation
Source: Sci Rep. 2016 Nov 8;6:36662. doi: 10.1038/srep36662 (PMC5099608; doi:10.1038/srep36662)
Supplement: Supplementary Information [file srep36662-s1.pdf]

Nitidine chloride prevents OVX-induced bone loss *via* suppressing NFATc1-mediated osteoclast differentiation

Qian Liu*, Tao Wang*, Lin Zhou, Fangming Song, An Qin, Hao Tian Feng, Xi Xi Lin, Zhen Lin, Jin Bo Yuan, Jennifer Tickner, Hua Gang Liu, Ming Hao Zheng, Jiake Xu [a], Jin Min Zhao [a]

* Equal contributors of the work

a Corresponding author:

Prof Jinmin Zhao, ZJinMin@hotmail.com, and Prof. Jiake Xu, jiake.xu@uwa.edu.au

1. Size of osteoclasts

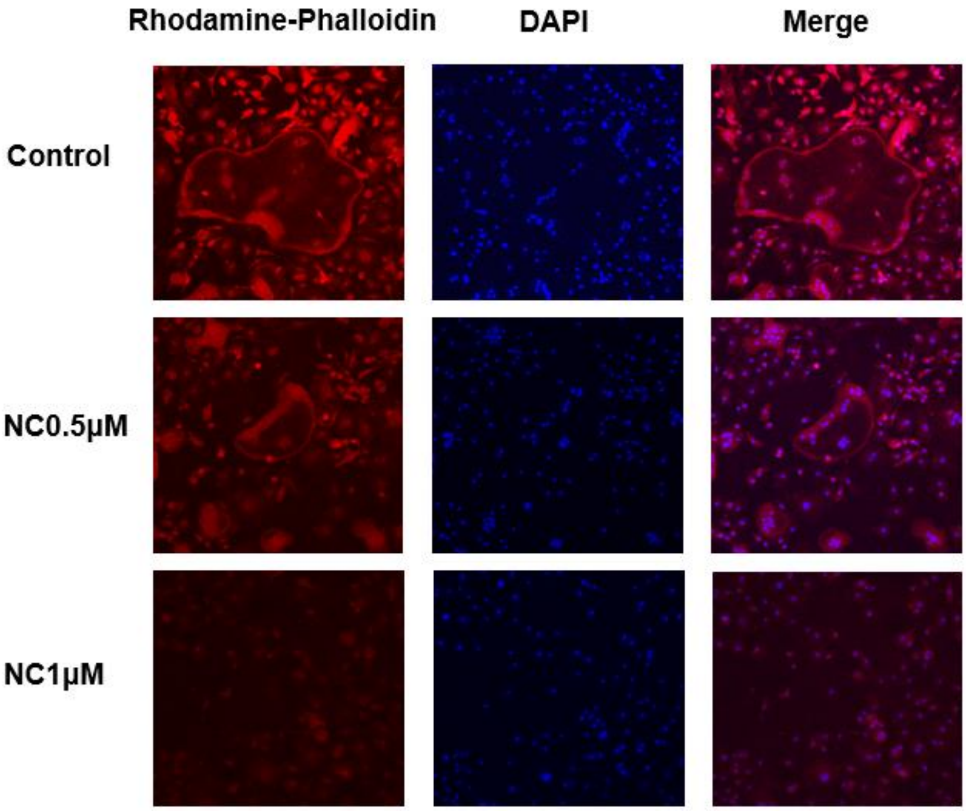

Supplementary Figure S1. **Nitidine chloride reduces the size of TRAP-positive multinuclear cells.** BMMs were cultured in the presence of RANKL with NC in 96-well plates. After 5 days of culture, cells were fixed with 4% paraformaldehyde and stained with Rhodamine-Phalloidin (F-actin structure) and DAPI (nuclear staining).

2. The protein level of IκBα

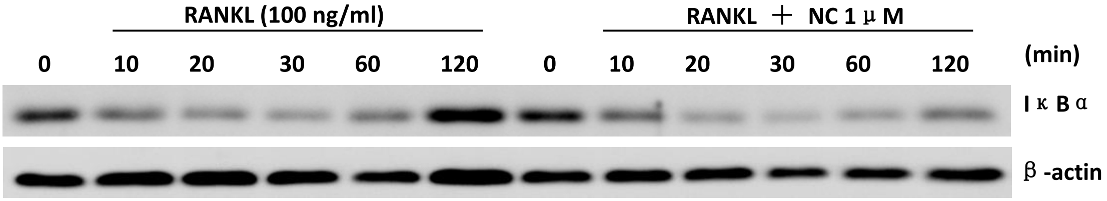

Supplementary Figure S2. **The effect of NC on RANKL-induced degradation of IκBα.** BMMs were pretreated with 1 μM NC for 1 hour, and then stimulated with RANKL for the indicated times. Cell lysates were prepared and subjected to western blotting with IκBα antibody. The β-actin blot is shown as loading control. Note that IκBα and β-actin protein blot slabs were cropped from full blots with the corresponding well established sizes of IκBα and β-actin proteins.

3. P65 nuclear translocation

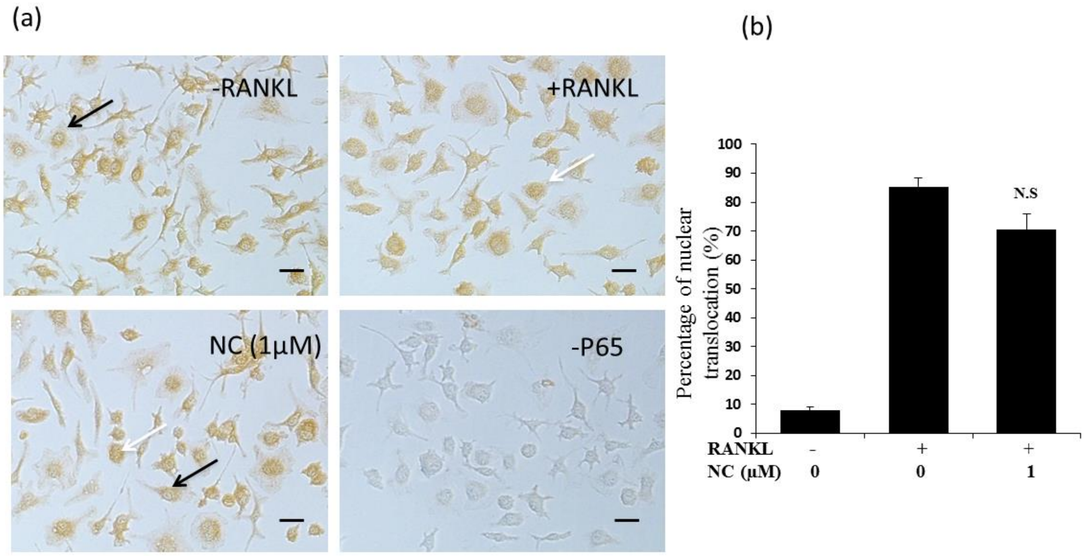

Supplementary Figure S3. **The effect of of Nitidine chloride on p65 nuclear translocation on BMMs.** (a) Light microscope images depicts the effect of NC on RANKL-induced P65 nuclear translocation. Black arrow presents P65 in cytoplasm while white arrow presents P65 in nucleus. Scale bar=50 μm. (b) The bar graph shows the percentage of cells showing nuclear translocation. Values are means ± SE from 3 repeated wells.
